# miR-302b inhibits tumorigenesis by targeting EphA2 via Wnt/ β-catenin/EMT signaling cascade in gastric cancer

**DOI:** 10.1186/s12885-017-3875-3

**Published:** 2017-12-22

**Authors:** Jin Huang, Yijing He, Howard L. Mcleod, Yanchun Xie, Desheng Xiao, Huabin Hu, Pan Chen, Liangfang Shen, Shan Zeng, Xianli Yin, Jie Ge, Li Li, Lanhua Tang, Jian Ma, Zihua Chen

**Affiliations:** 1 Department of Oncology, Xiangya Hospital, Central South University, Changsha, 410008 China; 2 Department of Dermatology, XiangYa Hospital, Central South University, Changsha, 410008 China; 3 Department of Clinical Pharmacology, XiangYa Hospital, Central South University, Changsha, 410008 China; 4 Hunan Key Laboratory of Pharmacogenetics, Changsha, 410008 China; 5 Moffitt Cancer Center, DeBartolo Family Personalized Medicine Institute, Tampa, FL 33612 USA; 6 Department of Pathology, Xiangya Hospital, Central South University, Changsha, 410008 China; 7 The Sixth Affiliated Hospital of Sun Yat-Sen University, Guangzhou, 510655 China; 8 Department of Hepatobiliary Surgery, Hunan Cancer Hospital and The Affiliated Cancer Hospital of Xiangya School of Medicine, Central South University, Changsha, 410013 China; 9 Department of gastroenterology and urology, Hunan Cancer Hospital and The Affiliated Cancer Hospital of Xiangya School of Medicine, Central South University, Changsha, 410013 China; 10 Department of General Surgery, Xiangya Hospital of Central South University, No.87 Xiangya Road, Changsha, 410008 People’s Republic of China; 11 Cancer Research Institute, Hunan Key Laboratory of Nonresolving Inflammation and Cancer, Central South University, No.138 Tongzipo Road, Changsha, China

**Keywords:** miR-302b, EphA2, Gastric cancer, Epithelial-mesenchymal transition, Wnt/β-catenin

## Abstract

**Background:**

EphA2 is a crucial oncogene in gastric cancer (GC) development and metastasis, this study aims to identify microRNAs that target it and serve as key regulators of gastric carcinogenesis.

**Methods:**

We identified several potential microRNAs targeting EphA2 by bioinformatics websites and then analyzed the role of miR-302b in modulating EphA2 in vitro and in vivo of GC, and it’s mechanism.

**Results:**

Our analysis identified miR-302b, a novel regulator of EphA2, as one of the most significantly downregulated microRNA (miRNA) in GC tissues. Overexpression of miR-302b impaired GC cell migratory and invasive properties robustly and suppressed cell proliferation by arresting cells at G0–G1 phase in vitro. miR-302b exhibited anti-tumor activity by reversing EphA2 regulation, which relayed a signaling transduction cascade that attenuated the functions of N-cadherin, β-catenin, and Snail (markers of Wnt/β-catenin and epithelial-mesenchymal transition, EMT). This modulation of EphA2 also had distinct effects on cell proliferation and migration in GC in vivo.

**Conclusions:**

miR-302b serves as a critical suppressor of GC cell tumorigenesis and metastasis by targeting the EphA2/Wnt/β-catenin/EMT pathway.

**Electronic supplementary material:**

The online version of this article (10.1186/s12885-017-3875-3) contains supplementary material, which is available to authorized users.

## Background

Gastric cancer (GC) is one of the most lethal malignancies worldwide [1, 2]. Recently, the Cancer Genome Atlas (TCGA) suggested four types of GC [3].The key driver genes in GC are still challenging to discern because the cancer has a high level of heterogeneity. Hence, there is an urgent need to better understand the detailed mechanisms that underline GC tumorigenesis and progression.

Receptor tyrosine kinases (RTKs) have emerged as key regulators in carcinogenesis among several solid tumors [4, 5]. EphA2 belongs to the family of RTKs and functions in bi-directional signal transduction via direct contact with adjacent cells expressing its specific ligand, EphrinA1. Usually, EphA2 is expressed at low levels in normal epithelial cells [6], whereas high levels of EphA2 have been observed in many solid tumors. Growing evidence indicate that EphA2 plays an important role in cellular transformation, primary tumor initiation, progression, and angiogenesis, and tumor invasion [7–9].

Recently, much attention has been focused on targeting EphA2 in the treatment of pediatric malignant glioma [10, 11]. We have previously demonstrated that EphA2 over-expression accelerated proliferative and metastatic properties in GC cells [12, 13], and promoted the epithelial-mesenchymal transition (EMT) through activating Wnt/β-catenin signaling [14]. Therefore, EphA2 is a proper candidate for developing targeted GC therapy that could inhibit metastasis and induce cytotoxicity in tumor cells while sparing normal cells. However, the regulation of EphA2 and cause of its overexpression in GC are still largely unknown.

miRNA usually negatively regulate gene expression primarily through interaction with the 3′-untranslated region (3’UTR) of target mRNAs [15]. miRNAs are well known to contribute to tumorigenesis in multiple ways in several cancers, including GC. Recent studies have identified regulatory activities of several miRNAs in GC cell growth, invasion, and migration [16–18], which supports that regulation of these miRNAs could potentially be developed into novel GC therapies.

In this study, we identified miR-302b as one of the most significantly downregulated miRNAs in GC cells. We show that miR-302b is a critical suppressor of GC cell growth and metastasis both in vitro and in vivo, and it inhibits downstream pathways (EMT and Wnt/β-catenin signaling) by directly targeting EphA2. Moreover, our results provide a potential epigenetic target for potential gastric cancer therapies that intervene with EphA2.

## Methods

### Antibodies and reagents

Primary antibodies for EphA2 (#6997, diluted1:1000), Snai1 (#3879, diluted1:1000), β-catenin (#8480, diluted1:1000), E-cadherin (#3195, diluted1:1000), N-cadherin (#13116, diluted1:1000), c-Myc (#13987, diluted1:1000), CyclinD1 (#2978, diluted1:1000), and GAPD- H (#2118, diluted1:1000) were purchased from Cell Signaling technology MA, USA.

### Cell culture

The human gastric adenocarcinoma cell line SGC-7901 was purchased from the Cell Resource Center of Xiangya Central Experiment Laboratory, Central South University (Changsha, Hunan, China) and cultured in RPMI 1640 medium (Hyclone, Waltham, USA). The human gastric adenocarcinoma cell line AGS was purchased from the American Type Culture Collection (Manassas, VA, USA) and cultured in F-12 K medium. All the cell lines were maintained with 10% fetal bovine serum (FBS) in a humidified incubator (37 °C, 5% CO_2_).

### Cell infection, transfection, and conditioned media preparation

Cell transfection with 2 μM of miRNA mimics or miRNA inhibitors and their negative controls (designed and synthesized by Genepharma [Shanghai,China]) was conducted using Lipofectamine 2000 (Invitrogen,Grand Island, NY, USA). The sequences for these miRNAs are listed in Additional file 1: Table S1.

The 3’UTR of EphA2 sequence was amplified and sub-cloned into the pMIR-REPORT luciferase vector (Ambion, Austin, TX, USA). Mutations in the seed region of the putative miRNA-binding sites of EphA2 mRNA were generated by point mutation PCR. All primers used for this purpose are described in Additional file 2: Table S2.

### MTT assay

Cells were seeded in 96-well plates at a density of 1 × 10^4^ cells per well and incubated for 24 h. After varying lengths of time, 10 μl of MTT dye (5 mg/ml, Sigma-Aldrich) was added to each well, and cells were incubated for another 4 h at 37 °C. Afterward, DMSO (150 μl) was added to each well and mixed for 10 min. Spectrometric absorbance at 490 nm was determined using a microplate reader (Bio-Rad, Hercules, USA). Each sample had three replicates.

### Cell cycle analysis

Cells were harvested 48 h after seeding, and single-cell suspensions containing 1 × 10^6^ cells were fixed with 75% alcohol ethanol. The cell cycle was monitored using propidium iodide (PI) staining of the nuclei. The fluorescence of DNA-bound PI in the cells was measured with a FACScan Flow Cytometer (BD Biosciences, San Diego, CA, USA) [14].

### Scratch wound-healing assay

Cells were plated and grown overnight to confluence in a 6-well plate. Monolayers of cells were wounded by dragging a pipette tip across the surface of the monolayer. Cells were washed to remove cellular debris and allowed to migrate for 24 h. Images were taken at 0 h and 24 h after wounding using an inverted microscope (Olympus, Japan) [14].

### Cell invasion assay

Transwell invasion assays were performed in 24-well, 8-μm pore size, transwell plates according to the manufacturer’s instructions (Corning, New York, NY, USA). The bottom of transwell chamber was coated with BD Matrigel Basement Membrane Matrix. The upper chamber was filled with 1 × 10^5^ cells in RPMI 1640 containing 5% FBS. The lower chamber was filled with RPMI 1640 containing 25% FBS as a chemo-attractant. After the chambers were incubated for 24 or 48 h at 37 °C, non-invading cells on the upper side of the chamber were removed from the surface of the membrane by scrubbing, and invading cells on the lower surface of the membrane were fixed with methanol, mounted, and dried. The number of cells invading through the matrigel was counted by a technician blinded to the experimental settings in four randomly selected microscopic fields of each filter. The test was conducted in three biological replicates.

### Western blot

Whole cell extracts were prepared using 0.14 M NaCl, 0.2 M triethanolamine, 0.2% sodium deoxycholate, 0.5% Nonidet P-40 and supplemented with a protease inhibitor (Sigma-Aldrich). Fifty micrograms of protein lysate was loaded into each well lysates were resolved by SDS-PAGE on 10% polyacrylamide gels, and then they were transferred to nitrocellulose or PVDF membranes. After blocking with 5% milk, the transferred membranes were subsequently incubated overnight at 4 °C with primary antibody, followed by secondary antibody for 1 h at routine temperature. Bands were visualized using an ECL Advance Detection System (Amersham Biosciences, Piscataway, NJ, USA).

### In vivo tumorigenesis

For in vivo studies, 1 × 10^6^ SGC-7901 cells stably expressing miR-302b, miR-NC, or no vector (wild-type) were injected subcutaneously into the flanks of male BALB/c nude mice at 5 weeks of age as previously described [19]. After 30 days, the mice were sacrificed, and tumor masses were measured. GC lung metastases were formalin-fixed, paraffin-embedded, and assessed by hematoxylin and eosin (H&E) staining. The experiments were performed using three mice per group, and all animal experiments were performed in strict accordance with the principles and procedures approved by the Committee on the Ethics of Animal Experiments of Central South University.

### Luciferase assay

The 3’-UTR sequence of EphA2 was amplified from normal human genomic DNA (NM_004431) and sub-cloned into the pmirGLO luciferase reporter vector (Promega). SGC-7901 or AGS cells at 70–80% were co-transfected with wild-type (WT) or mutant (Mut) 3’-UTR vectors and miR-302b-3p mimics or inhibitors using Lipofectamine 2000. At 48 h post-transfection, the cells were assayed for luciferase activity using the Dual-Luciferase Reporter Assay System (Promega) according to the manufacturer’s instructions. Firefly luciferase activity in each sample was normalized to Renilla luciferase activity. The firefly luciferase activity of the cells that were transfected with miRNA mimics or inhibitors is represented as the percentage of activity relative to that of cells that were transfected with negative controls. All experiments were performed in triplicate.

### Statistical analysis

Results are expressed as mean ± SEM from at least three independent experiments. Using the GraphPad Prism statistical program, data were analyzed using Student’s *t*-test, unless otherwise specified (Mann–Whitney test, ROC, Pearson correlation, etc.). Statistical analyses were performed using IBM SPSS22.0 software (SPSS). Two-tailed *P* < 0.05 was considered to be statistically significant.

## Results

### miR-302b is a novel repressor targeting EphA2 directly in GC

**Fig. 1:**
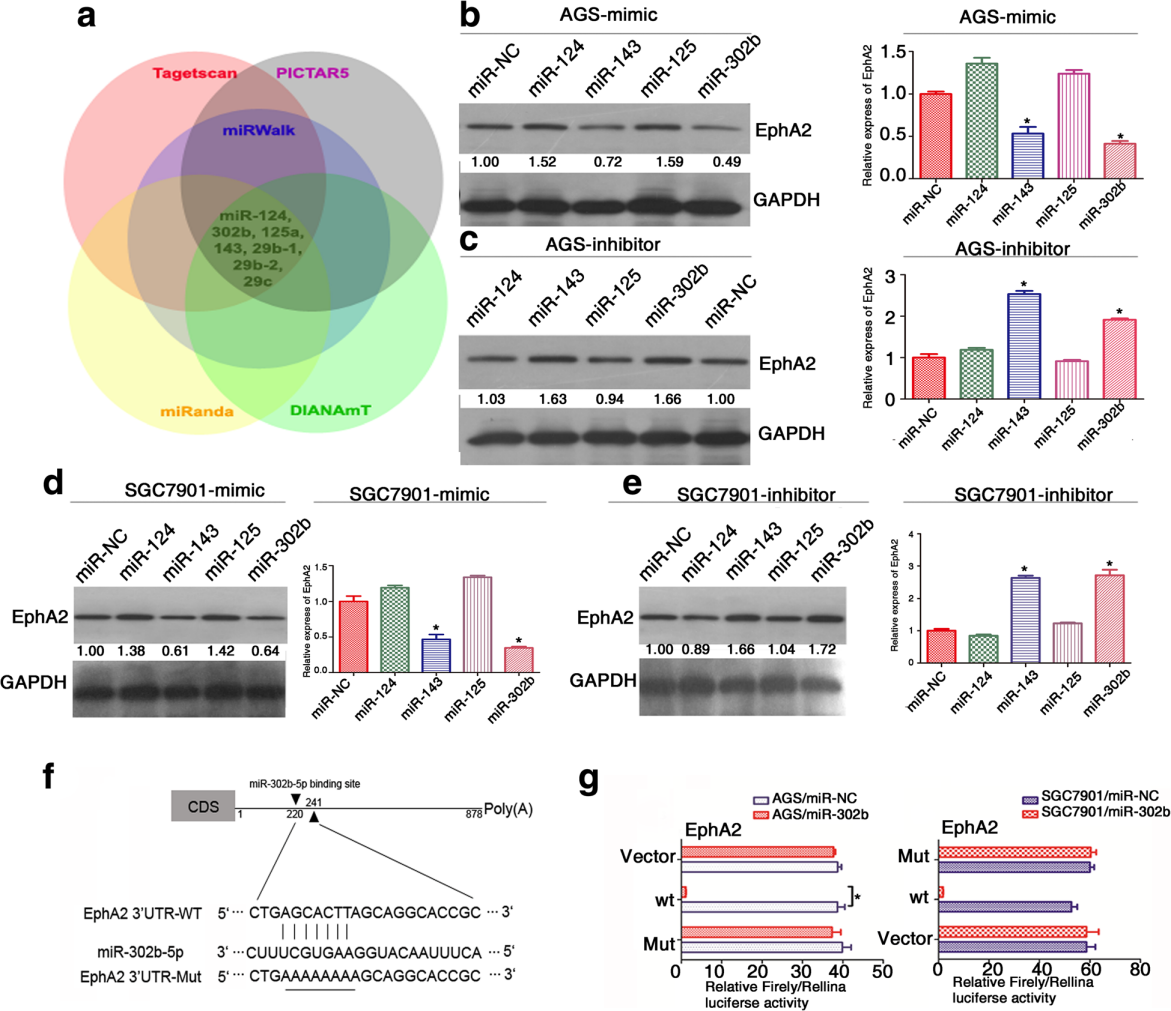
miR-302b inhibits EphA2 mRNA and protein levels in gastric cancer cells. **a** The diagram shows the bioinformatic prediction and initial screening of candidate miRNAs that may target EphA2.miRNA mimics and inhibitors were transfected into AGS (**b**, **c**) and SGC7901(**d**, **e**). The expression of EphA2 was determined by qRT-PCR and western blot(for qRT-PCR, the expression values of “mimics or inhibitor control” were set to 1). GAPDH was used as the internal control. **f** miR-302b downregulates EphA2 expression through specifically its 3’UTR. **g** Luciferase activity of WT 3’UTR-EphA2-luc and mutant 3’UTR-EphA2-luc constructs in AGS, SGC7901 cells after transfection of miR-302b plasmid. *P < 0.05

We conducted a bioinformatic search for potential miRNAs targeting the mRNA of EphA2 using five miRNA databases: miRWalk, DIANAmT, miRanda, PICTAR5, and Targetscan. We identified 60 miRNAs, among which six miRNAs (miR-200a/miR-200b [20, 21], miR-26a/ miR-26b [22], miR-141 [17], miR-520d-3p [19]) had been reported to target EphA2 in a few tumor types. Of note, seven of the 60 identified miRNAs were dysregulated in GC according to previous studies: miR-124 [23], miR-302b [24], miR-125a-5p [25], miR-143 [26, 27], miR-29b-1/miR-29b-2/miR-29c [27, 28]. Thus, we focused on these seven miRNAs in subsequent analyses (Fig. 1a) as candidate miRNAs involved in regulating EphA2 expression in GC.

Among these differentially expressed miRNAs in GC cell lines AGS and SGC7901, miR-302b and led to a decreased EphA2 expression, however we chose miR-302b for further investigation because following results preferred miR-302b (data not shown). miR-141 and miR-26b served as a positive control. Other miRNAs resulted upregulation of EphA2 at both mRNA and protein levels (Fig. 1b-e). Because the role of miR-302b in GC has not been fully documented, we thus chose miR-302b for further investigation as a regulator of EphA2 in GC.

To further validate EphA2 as a target of miR-302b, we cloned the full length EphA2 3’UTR (878 bp) into a luciferase reporter, as shown in Fig. 1f. We found that miR-302b overexpression substantially repressed activity of the reporter that carried WT, but not mutant, EphA2–3’UTR, suggesting that the regulation is mediated in sequence-specific manner and the tested region is a bonafide miR-302b-targeting site (Fig. 1g). These data suggest that miR-302b is a novel negative regulator that targets EphA2 directly in GC.

### miR-302b inhibits GC cell proliferation and cell cycle progression by targeting EphA2

**Fig. 2:**
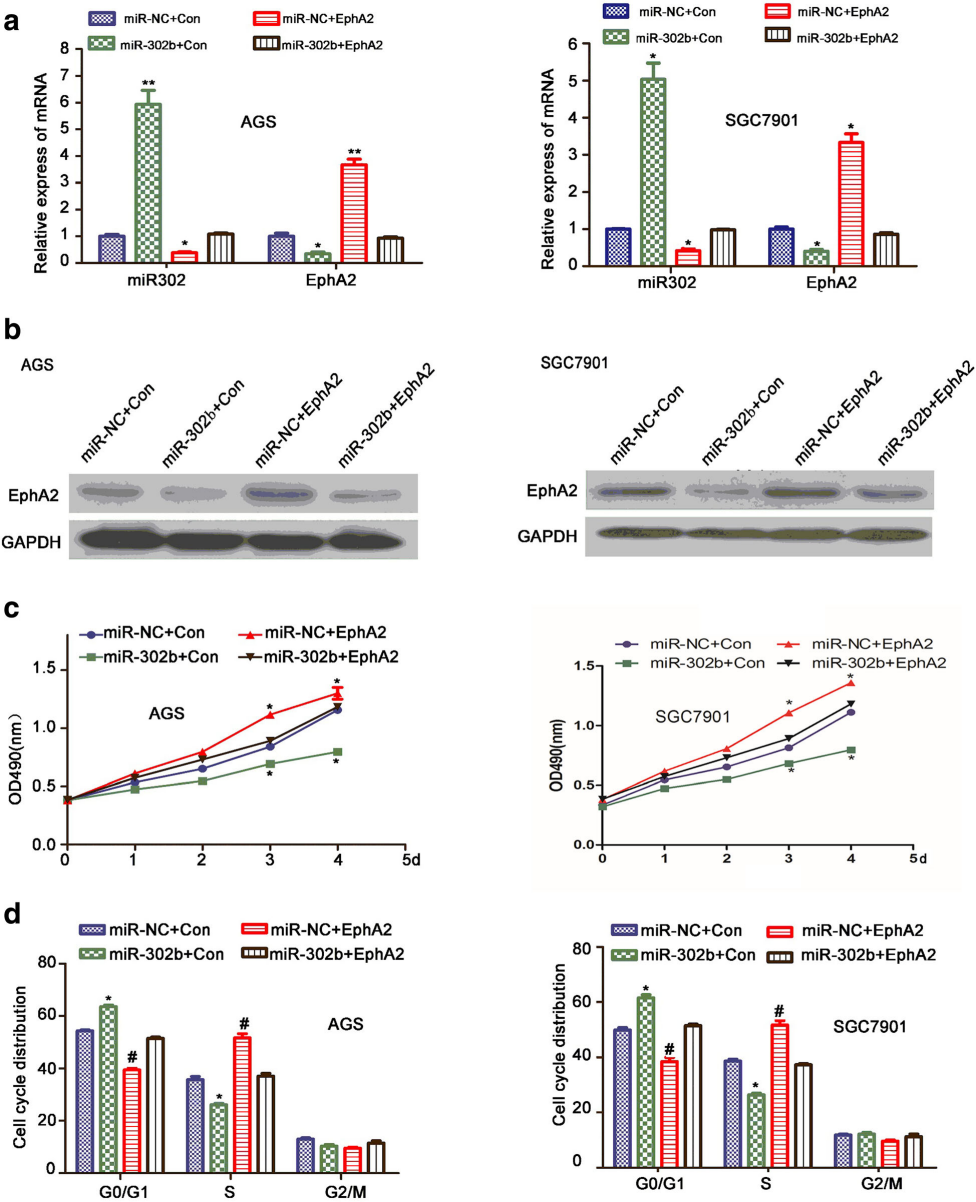
miR-302b suppresses cell proliferation and cell cycle arrest in vitro by targeting EphA2. **a** miR-302b and EphA2 expression in cells overexpressing miR-302b or co-expressing miR-302b and EphA2 by real-time PCR. **b** EphA2 expression in cells overexpressing miR-302b or co-expressing miR-302b/EphA2 by Western blot. **c** Effects of miR-302b and miR-302b/EphA2 co-expression on cell proliferation by MTT assay. **d** Flow cytometry analysis of cell cycle distribution in cells overexpressing miR-302b or co-expressing miR-302b and EphA2. The bar graph displays the percentage of cells in phases G0-G1,S, and G2. *P < 0.05, ^#^ *P* < 0.01

To explore the functional significance of miR-302b in tumor progression in GC cells, AGS and SGC-7901 cells were transfected with 1) a miR-302b mimic or miR-NC,2) aEphA2 expression vector or control, or 3) co-transfected with the miR-302b mimic and EphA2 expression vector or controls. As the results show in Fig. 2a and b, the mRNA and protein expression of EphA2 were clearly reduced bymiR-302b mimic transfection.

Cell proliferation was determined at 24, 48, 72, and 96 h using an MTT(3-(4,5-dimethythiazol-2-yl)-2,5-diphenyltetrazolium bromide) assay. As shown in Fig. 2c, the proliferation of SGC-7901 and AGS cells was significantly increased upon EphA2overexpression, and decreased whenmiR-302b was present, at each time point. The proliferation curves indicated that enhanced proliferation of GC cells caused by EphA2 could be reduced through expression of miR-302b.

Cell cycle phase distribution was determined by flow cytometry analysis. Figure 2d shows that, similar to our previous research, EphA2 overexpression resulted in a substantial reduction in the number of cells in G0–G1 phase and an increase in the number of cells in S phase at 48 h after seeding. Little change was observed for G2–M phase. Overexpression of miR-302b had the opposite effect, increasing the number of cells in G0–G1 phase and reducing the number of cells in S phase. We also observed that miR-302b inhibited the EphA2-overexpression-induced increase in S phase cells. These results suggest that miR-302b may inhibit GC cell cycle progression in vitro by targeting EphA2.

### miR-302b inhibits GC invasion and metastasis in vitro and in vivo

**Fig. 3:**
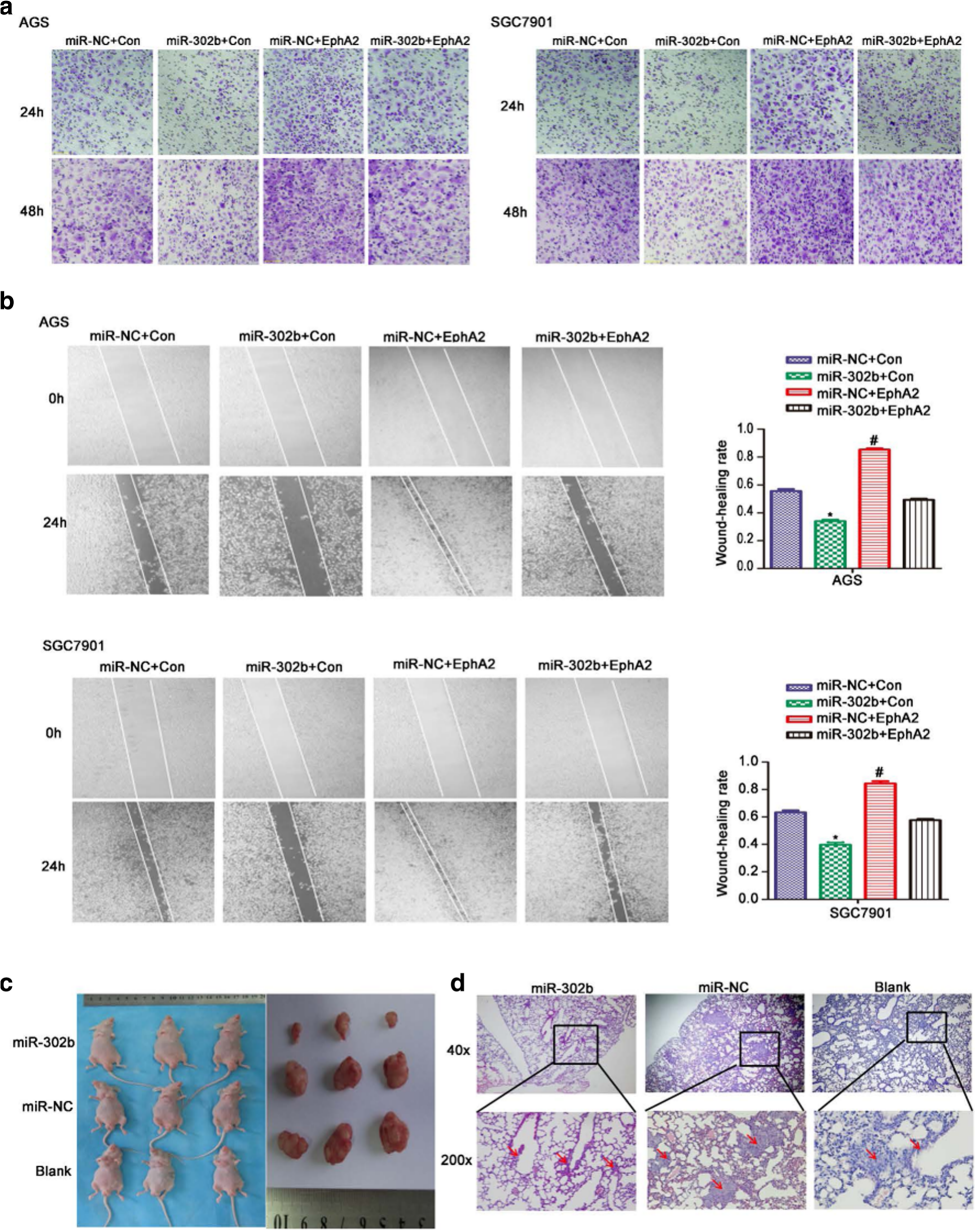
Overexpression of miR-302b attenuates the tumor growth and metastasis properties of GC cells in vitro and in vivo. **a** The transwell migration, and (**b**) Wound-healing, assays demonstrated the effects of miR-302b and miR-302b/EphA2 on cell invasion.All data are shown as the mean ± SEM. *P<0.05 relative to control. **c** Tumor growth in mouse xenograft model. SGC7901 cells (“blank,” “miR-NC,” or“miR-302b”) were injected subcutaneously into nude mice. After 30 days, the mice were sacrificed, and images of whole tumors were acquired. **d** H&E stained sections of lungs isolated from nude mice receiving atail vein injection of SGC7901 cells. Original magnification, 40× or 200×. ^#^ *P* < 0.01

To determine whether miR-302b influence on EphA2 affects the migration and mobility of GC cells, an in vitro cell invasion assay was performed based on the principles of the Boyden chamber assay. Cells that migrated through the Matrigel matrix are presented in Fig. 3a. When endogenous EphA2 was inhibited by miR-302b in the cells co-expressing miR-302b and EphA2, the number of cancer cells migrating through the Matrigel decreased significantly compared with the EphA2 overexpression group, while miR-302b over-expression group showed the opposite effect comparing with the control group (*P* < 0.05). To provide further support for the direct effect of miR-302b targeting EphA2 on cell migration ability, we used an in vitro wound-healing assay. As shown in Fig. 3b, the miR-302b/EphA2 co-expressing GC cells migrated remarkably slower thanEphA2 overexpressing cells (*P* < 0.05). These results implied that the ability of EphA2 to facilitate GC cell migration and invasion could be suppressed by miR-302b.

Our previous results in nude mice that had received tail vein injection of EphA2-overexpressing GC cells demonstrated that EphA2 overexpression could give rise to metastases in multiple organs [14]. Here, we sought to study the effects of miR-302b in this in vivo model. We used SGC7901 cells stably transfected with miR-302b for xenografts in nude mice. The nude mice transplanted with SGC-7901 cells stably overexpressing miR-302b or miR-NC/blank developed solid tumors within 30 days. As shown in Fig. 3c, tumor volume and weight were significantly smaller in mice received a xenograft of miR-302b-expressing SGC7901s than in mice xenografted with control cells (miR-NC or blank SGC-7901 cells). We also explored whether miR-302b could inhibit GC cell metastasis in vivo. We injected nude mice with SGC-7901 cells (blank, miR-NC, miR-302b) via the tail vein and quantified metastatic foci in the lungs after 30 days. We observed fewer metastatic nodules in mice receiving miR-302b-overexpressing cells than those receiving control cells (Fig. 3d).

### Downregulation of EphA2 by miR-302b suppressed EMT in GC cells

**Fig. 4:**
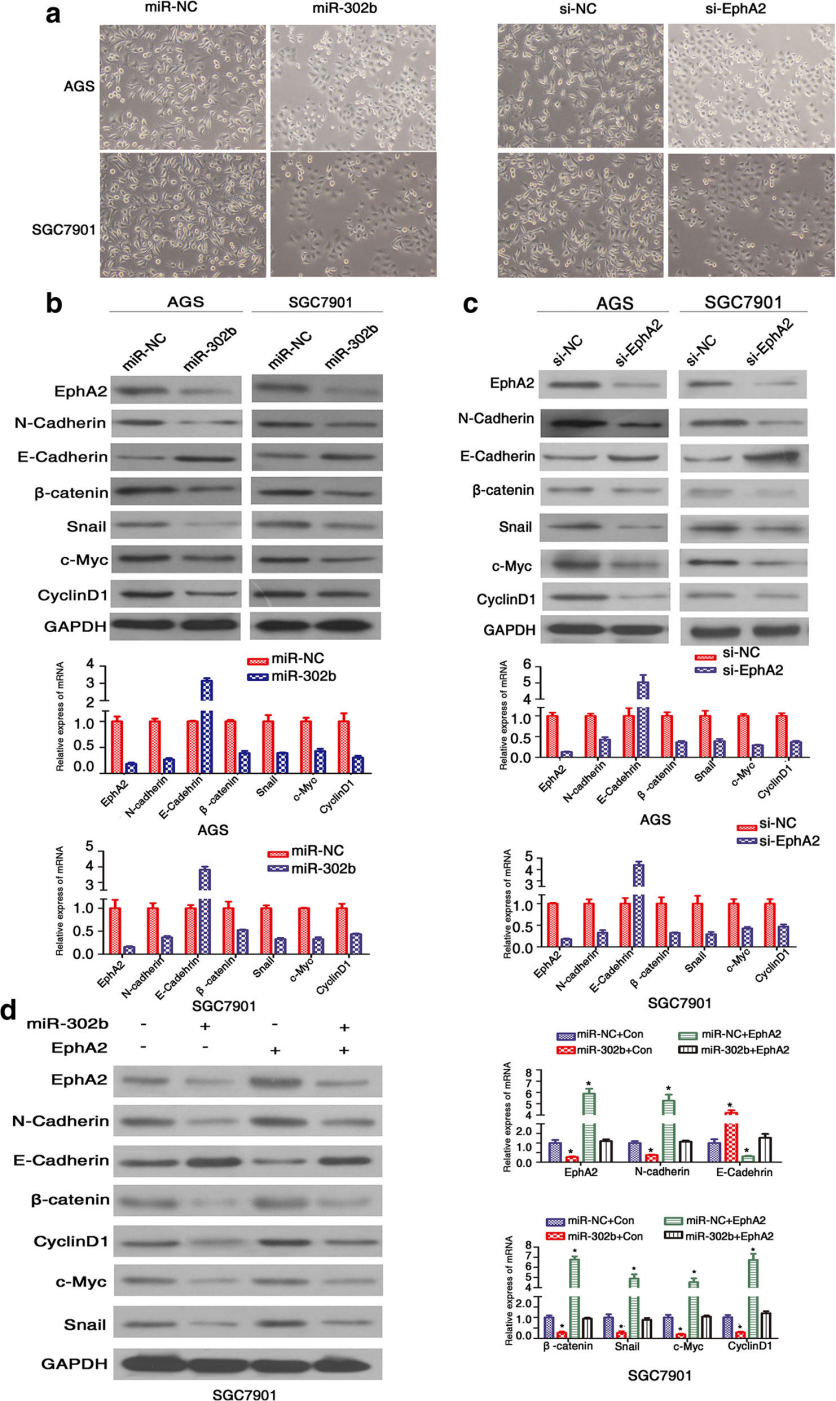
Ectopic miR-302b expression modulates EMT markers and the Wnt/β-catenin pathway by regulating expression of EphA2. **a** miR-302b and EphA2 siRNA(si-EphA2) reduced morphological characteristics of EMT. **b** Western blot (left) andqRT-PCR(right) analyses showing decreased levels of N-cadherin, β-catenin,Snail, c-Myc, and Cyclin D1, as well as increased E-cadherin, in miR-302b-overexpressed AGS and SGC7901 cells, as compared with the miR-NC counterparts. **c** qRT-PCR(right) and western blot(left) analysis for N-cadherin, E-cadherin,β-catenin,Snail, c-Myc, and Cyclin D1 following transfection with EphA2 siRNA (si-EphA2) versus control(si-NC). **d** The mRNA and protein levels of N-cadherin,E-cadherin, β-catenin,Snail,c-Myc,CyclinD1 were determined by qRT-PCR and western blot analysis after co-transfection with miR-302b(or a control) and EphA2 (or a control) expression vectors in AGS and SGC7901 cells. β-actin served as an internal control

Research has suggested that reprogramming of the EMT has anti-tumor benefits, including decreased tumorigenesis and invasion, and thus favors uncontrolled tumor cell growth and metastasis [14]. In our previous study, we showed that EphA2 promotes the EMT in GC cells. Some evidence shows that miR-302bregulates the development of death receptor resistance and EMT progression in breast cancer [29]. We reasoned that miR-302b might play a significant role in EMT by targeting EphA2 in GC. To test this hypothesis, we examined cultures of AGS and SGC-7901 cells overexpressing miR-302b, with or without overexpressing EphA2 (all by transient transfection assay). We looked for morphological characteristics of EMT, which include the loss of epithelial characteristics and acquisition of mesenchymal properties, such as spindle-like fusiform shape and loss of sheet-like architecture in epithelial cells [30]. We observed that miR-302b-overexpressing cells and si-EphA2 cells possessed fewer mesenchymal-like properties than miR-NC-expressing cells (Fig. 4a). E-cadherin, Snail, N-cadherin, and β-catenin are well-known, specific markers for the EMT process. We quantitated the expression levels of these markers by real-time PCR and Western blot analysis in AGS and SGC-7901 cells overexpressing miR-302b (Fig. 4b and c). Compared with the miR-NC group, the mRNA and protein levels of Snail, N-cadherin, and β-catenin decreased significantly in miR-302b-overexpressing cells. Inversely, the expression level of E-cadherin increased 4.3-fold. Additionally, we observed that miR-302b expression downregulated expression of Snail, N-cadherin and β-catenin by suppressing EphA2 expression, while upregulating expression of E-cadherin (Fig. 4d). These results suggest that miR-302b is a suppressor force of the EMT by modulating EphA2 in GC cells.

### miR-302b regulates Wnt/β-catenin pathway

Previously, we reported that EphA2activated the Wnt/β-catenin pathway in GC [14]. Therefore, we extended our studies on miR-302b and EphA2 to look at effects further down- stream. We used real-time PCR and Western blot to measure downstream targets, such asCyclin-D1 and c-Myc. As shown in Fig. 4b and c, overexpression of miR-302b led to significantly reduction of Cyclin-D1 and c-Myc mRNA and protein relative to control groups, results that were similar to those be seen in EphA2 silenced cells. The data also showed that mRNA and protein expression of Cyclin-D1 and c-Myc were reduced sharply in cells co-transfected with miR-302b and EphA2, presumably by miR-302b downregulation of EphA2expression (Fig. 4d). These results confirmed that miR-302b might act as a tumor suppressor by negatively regulating the EphA2/Wnt/β-catenin pathway in GC cells.

## Discussion

Identification of crucial molecular determinant(s) is essential to find alternative strategies to overcome the current bottleneck in tumor therapies, including those for GC. As a member of RTK family, it is well known that EphA2 is an emerging target for cancer therapeutics, due to its increased expression in tumor tissues [12–14]. Downregulation of EphA2 expression through various approaches inhibits malignant behavior in vitro and in vivo [31–33]. To date, the knowledge of functional roles and regulatory mechanism of EphA2 in GC are remains unclear due to the lack of applicable and practicable method to target it.

Recent studies have indicated that dysregulation of miRNAs results in dysregulated EphA2 in several kinds of solid tumors, including prostate, breast, and colon cancers [27, 28, 34]. Among these reported microRNA profiles and online predicted results, we found seven miRNAs (miR-124, miR-302b, miR-125a-5p, miR-143, miR-29b-1, miR-29b-2, miR-29c) that were predicted to target EphA2 in GC. Further experiments demonstrated that miR-302b was the most significant downregulating miRNA of EphA2 in GC cells. Thus, we focused our studies on miR-302b and its role in regulating EphA2 in GC. Our results establish EphA2 as a novel, direct, functional effector of miR-302b in GC. This finding is supported by the observation that the overexpression of miR-302b in GC cells markedly downregulated EphA2 expression through targeting with the 3’UTR of EphA2 mRNA, and vice versa.

MiR-302b, an embryonic stem cell (ESC)-specific microRNA, has been documented to regulate the EMT, which endows tumor cells with the ability to leave the primary tumor and invade the local tissue for metastatic spread [29, 35]. Loss of epithelial-cell markers (e.g., E-cadherin) and gain of mesenchymal-cell markers (e.g., N-cadherin, Snail), are reliable markers of EMT, particularly at the leading edge or invasion front of human solid tumors [36, 37]. It’s reported that miR-302b expression is lower in gastric cancer, and it can regulate cell proliferation and cell cycle through different pathways [24, 38–40]. Recently several reports, including our research in GC, have suggested that EphA2 may function as an activator of EMT in carcinogenesis [14, 41]. Here, we showed that miR-302b modulates EphA2-associated EMT changes in GC cells. This result is supported by the observation that miR-302b overexpression reduced the transformation of EphA2-mediated mesenchymal-like phenotype in GC cells. Our in vitro studies further showed that miR-302b also dramatically suppressed N-cadherin, Snail, and β-catenin expression levels, the markers of EMT, by regulating the expression of EphA2.

Because of the complexity of EMT induction in the tumor microenvironment, the EMT involves several related signaling pathways, such as tumor growth factor-β, nuclear factor-κB, Notch, and Wnt/β-catenin [42]. Wnt/β-catenin signaling has a major impact on EMT [43] during cancer progression. After Wnt receptor on the cell membrane is stimulated by the signal, promoting the translocation of β-catenin into the nucleus may result in the loss of E-cadherin and subsequent induction of EMT [44–46]. Inhibition of Wnt/β-catenin signaling can block EMT transcription factors and promote epithelial differentiation. We previously demonstrated that EphA2 regulated the EMT through Wnt/β-catenin signaling. Consistently, we found that miR-302b affects downstream Wnt/β-catenin signaling via EphA2.This result was confirmed by that EphA2 downregulation by miR-302b also decreased expression of c-Myc and CyclinD1, specific target oncogenes of the Wnt/β-catenin signal pathway. This is the first study to report this mechanism of miR-302b involvement in the EMT.

miR-302b acts as a tumor suppressor in several types of tumors by inhibiting cell growth and migration, and by modulating the cell cycle [47–49]. Several studies have demonstrated that reprogramming of the EMT promotes tumorigenesis and invasiveness, which favors uncontrolled tumor cell growth and metastasis [50, 51]. Our in vitro data suggest that miR-302b overexpression can attenuate GC cell migration and invasiveness, suppress cell proliferation, and induce cell cycle arrest in G0–G1 phase. Further research showed that these effects of miR-302b on GC cells are due to phenocopied changes in EphA2. Moreover, our in vivo studies using subcutaneous xenograft and tail vein injection models in mice indicates that overexpression of miR-302b led to a pronounced decrease in tumor volume and lung metastases. Based on these results, we posit that miR-302b could reduce GC proliferation and progression both in vitro and in vivo by immediately targeting EphA2-induced EMT through Wnt/β-catenin signaling.

## Conclusion

**Fig. 5:**
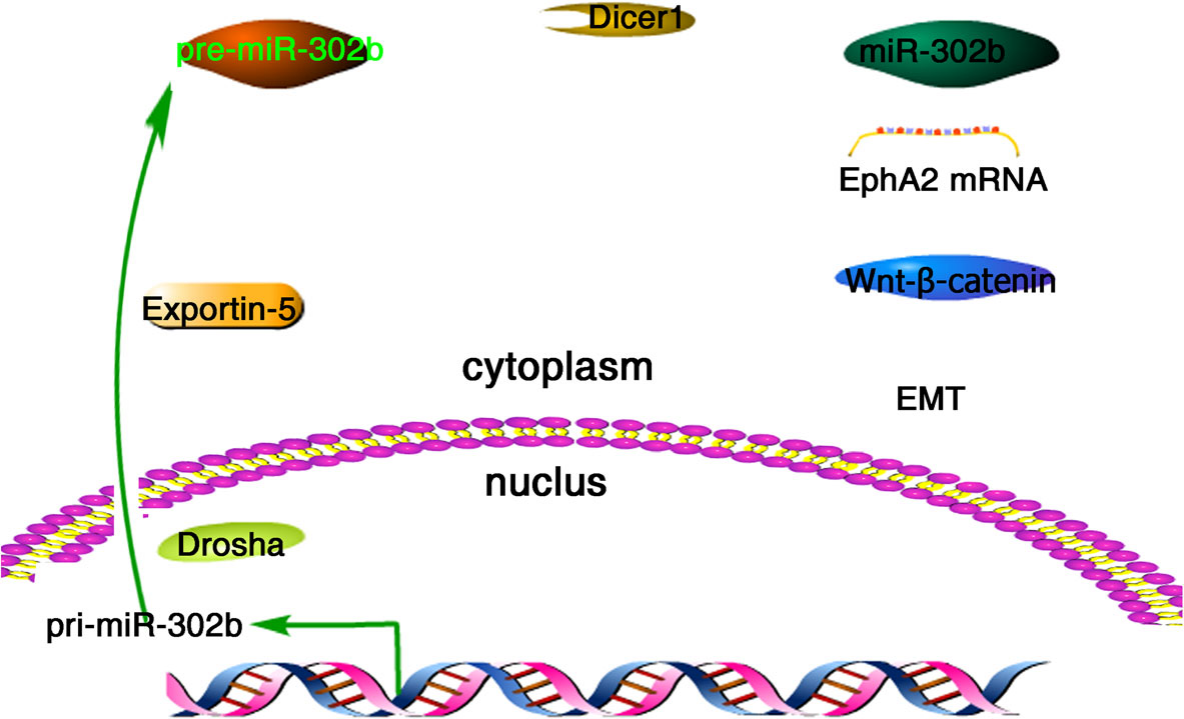
Proposed mechanism for miR-302b modulating gastric cancer proliferation, invasion and EMT via targeting EphA2

We demonstrated that miR-302b serves as a critical tumor suppressor of GC cell proliferation, invasion, and migration by modulating the EphA2/Wnt/ β-catenin/EMT signaling cascade (Fig. 5). These data provide novel insights into GC progression and offer miR-302b and EphA2 as potential prognostic factors and therapeutic targets for this disease.

## Additional files

Additional file 1: Table S1. The Sequences of the Candiate miRNAs involved in EphA2 regulating GC. (DOCX 17 kb)

Additional file 2: Table S2. The Primer Sequences for wild-type and mutant type of EphA2–3’UTR. (DOCX 17 kb)

## Abbreviations

EMT: Epithelial-mesenchymal transition
ESC: embryonic stem cell
FBS: Fetal bovine serum
GC: Gastric cancer
H&E: Hematoxylin and eosin
HR: Hazard ratio
miRNA: MicroRNAs
Mut: Mutant
OS: Overall survival
RTKs: Receptor tyrosine kinases
TCGA: The Cancer Genome Atlas
WT: Wild-type
