# Supplementary material for: miR-302b inhibits tumorigenesis by targeting EphA2 via Wnt/ β-catenin/EMT signaling cascade in gastric cancer
Source: BMC Cancer. 2017 Dec 22;17:886. doi: 10.1186/s12885-017-3875-3 (PMC5741943; doi:10.1186/s12885-017-3875-3)
Supplement: Supplementary file 1 — The Sequences of the Candiate miRNAs involved in EphA2 regulating GC. (DOCX 17 kb) [file 12885_2017_3875_MOESM1_ESM.docx]

**Supplemental table 1. The Sequences of the Candiate miRNAs involved in EphA2 regulating GC.**

| miRNA Name | The Sequences of miRNA |
| --- | --- |
| miR-302b-5p | 5’-ACUUUAACAUGGAAGUGCUUUC-3’ |
| miR-141-5p | 5’-CAUCUUCCAGUACAGUGUUGGA-3’ |
| miR-26b-5p | 5’-UUCAAGUAAUUCAGGAUAGGU-3’ |
| miR-124-3p | 5’-UAAGGCACGCGGUGAAUGCC-3’ |
| miR-125a-5p | 5’-UCCCUGAGACCCUUUAACCUGUGA-3’ |
| miR-143-3p | 5’-UGAGAUGAAGCACUGUAGCUC-3’ |
| miR-29b-3p | 5’-UAGCACCAUUUGAAAUCAGUGUU-3’ |
| miR-29c-3p | 5’- UAGCACCAUUUGAAAUCGGUUA-3’ |
