# Supplementary material for: miR-302b inhibits tumorigenesis by targeting EphA2 via Wnt/ β-catenin/EMT signaling cascade in gastric cancer
Source: BMC Cancer. 2017 Dec 22;17:886. doi: 10.1186/s12885-017-3875-3 (PMC5741943; doi:10.1186/s12885-017-3875-3)
Supplement: Supplementary file 2 — The Primer Sequences for wild-type and mutant type of EphA2–3’UTR. (DOCX 17 kb) [file 12885_2017_3875_MOESM2_ESM.docx]

**Supplemental table 2. The Primer Sequences for wild-type and mutant type of EphA2-3’UTR.**

| Primer | Sequence |
| --- | --- |
| Wild-type for ward primer | GCAAGCTTGCCTCGACAGGGCCT |
| Wild-type for reverse primer | CGACGCGTTCGGTTTGAATCATCTG |
| Mutational type for ward primer | GCGACGCACACAGCTGAAAAAAAAGCAGGCACCGCCACGGC |
| Mutational type for reverse primer | GCCGTGGCGGTGCCTGCTTTTTTTTCAGCTGTGCGTCGC |
